# Excited state dynamics of azanaphthalenes reveal opportunities for the rational design of photoactive molecules

**DOI:** 10.1038/s42004-024-01403-z

**Published:** 2025-01-09

**Authors:** Malcolm Garrow, Lauren Bertram, Abi Winter, Andrew W. Prentice, Stuart W. Crane, Paul D. Lane, Stuart J. Greaves, Martin J. Paterson, Adam Kirrander, Dave Townsend

**Affiliations:** 1 Institute of Chemical Sciences, Heriot-Watt University, Edinburgh, UK; 2 Physical and Theoretical Chemistry Laboratory, Department of Chemistry, University of Oxford, Oxford, UK; 3 Institute of Photonics & Quantum Sciences, Heriot-Watt University, Edinburgh, UK; 4 Present Address: Department of Chemistry, Brown University, Providence, RI USA

**Keywords:** Chemical physics, Reaction kinetics and dynamics, Photochemistry

## Abstract

Various photoactive molecules contain motifs built on aza-aromatic heterocycles, although a detailed understanding of the excited state photophysics and photochemistry in such systems is not fully developed. To help address this issue, the non-adiabatic dynamics operating in azanaphthalenes under hexane solvation was studied following 267 nm excitation using ultrafast transient absorption spectroscopy. Specifically, the species quinoline, isoquinoline, quinazoline, quinoxaline, 1,6-naphthyridine, and 1,8-naphthyridine were investigated, providing a systematic variation in the relative positioning of nitrogen heteroatom centres within a bicyclic aromatic structure. Our results indicate considerable differences in excited state lifetimes, and in the propensity for intersystem crossing *vs* internal conversion across the molecular series. The overall pattern of behaviour can be explained in terms of potential energy barriers and spin-orbit coupling effects, as demonstrated by extensive quantum chemistry calculations undertaken at the SCS-ADC(2) level of theory. The fact that quantum chemistry calculations can achieve such detailed and nuanced agreement with experimental data across a full set of six molecules exhibiting subtle variations in their composition provides an excellent example of the current state-of-the-art and is indicative of future opportunities for rational design of photoactive molecules.

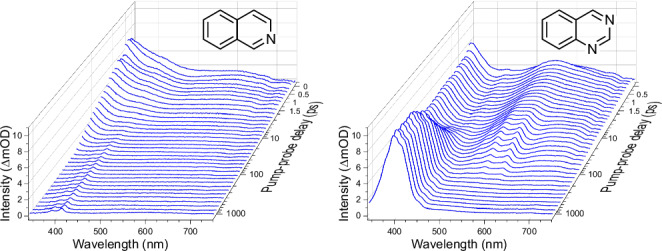

## Abstract (web summary)

Aza-aromatic heterocycles are interesting motifs present in various photoactive molecules, however, their rational design remains challenging due to the lack of details about their excited state photophysics and photochemistry. Here, the authors combine experimental and theoretical studies of UV photoexcitation and associated relaxation dynamics for six azanaphthalenes exhibiting compositional variations, interrogating the difference in excited state lifetimes and the propensity for intersystem crossing vs internal conversion across the molecular series.

## Introduction

Aromatic heterocycles containing nitrogen atoms (aza-aromatics) are common motifs in many biomolecules, with the most well-known instance being the purine and pyrimidine architectures within the DNA bases. Other examples include various alkaloids, the amino acids tryptophan and histidine, as well as folate (vitamin B_9_) and other structurally related pterin species that play important roles as enzyme cofactors and natural colourants^1–3^. Many industrial processes make use of aza-aromatic heterocycles, including the production of dyes and pigments, or pharmaceuticals^4,5^, and there is growing interest in their use for optoelectronics^6,7^ and organic solar cells^8^. Additionally, pterin derivatives are considered as photosensitizers for photodynamic therapies, as they exhibit large intersystem crossing (ISC) quantum yields and long-lived triplet states^9^. Given the widespread natural occurrence of aza-aromatic species and the range of potentially useful applications for novel synthetic variants, it is important to develop more detailed understanding of their excited-state photophysics and photochemistry. This has immediate relevance to any potential environmental impacts and medical treatments, and addresses fundamental questions relating to the ultraviolet (UV) photostability of many biomolecules^10^—potentially helping to prove a rationale for their evolutionary selection.

**Fig. 1:**
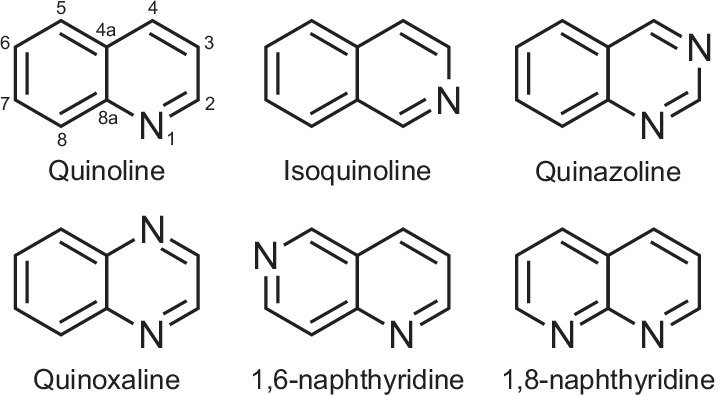
Schematic chemical structures of the six azanaphthalene molecules considered in this study. Quinoline and isoquinoline have a single N centre at two distinct positions in the ring, quinazoline and quinoxaline have two N centres at distinct positions in the same ring, and 1,6-naphthyridine and 1,8-naphthyridine have two N centres with one N atom per ring. Position numbering around the ring system is also indicated, which is referenced in parts of the Discussion.

We report a combined experimental and theoretical study of UV photoexcitation and associated relaxation dynamics across six nitrogen-containing bicyclic aromatic compounds shown in Fig. 1: the azanaphthalene species quinoline, isoquinoline, quinazoline, quinoxaline, 1,6-naphthyridine and 1,8-naphthyridine. These molecules have one or two N atoms distributed across the two rings. Of particular interest is how the relative position of these N atom centres influences the competing pathways for excess energy redistribution. This links to ongoing and extensive efforts towards rational design of photoactive molecules for a wide range of applications such as photocatalysis^11^, photosensitizers^12^, photovoltaics^13,14^, photochromic molecules^15^ and UV filters^16^. In this regard, bicyclic species provide more potential for systematic variation than their related monocyclic counterparts (e.g. pyridine, pyrazine, pyrimidine). Our study excludes diazanaphthalene species with direct N–N bonds (e.g. cinnoline, phthalazine) since such structures are not typically observed in nature, something likely arising due to a lack of naturally occurring hydrazine species, which are the common building blocks for their synthesis.

Given the long history of scientific studies involving the molecules considered here, we briefly review only the most relevant previous work. Building on an early study of quinoline and isoquinoline^17^, the excited-state photochemistry of azanaphthalenes began to attract considerable attention in the 1960s and 70s, with many investigations employing flashlamp excitation of species held within solid hydrocarbon matrices^18–23^. These studies noted the absence of significant fluorescence decay and determined the extinction coefficients for the triplet–triplet absorption bands of quinoline, isoquinoline and quinoxaline. Quantum yields for the competition between ISC and internal conversion (IC) back to the S_0_ ground state were also reported. Of interest here is the increased propensity for ISC observed in quinoline (*Φ*_ISC_ = 0.43–0.50) over isoquinoline and quinoxaline (*Φ*_ISC_ = 0.18–0.27). Numerous early spectroscopic studies indicate that in most azanaphthalenes the lowest-lying S_1_ singlet excited state is of nπ* character^24^, exhibiting weak absorption with origin onset thresholds above 325 nm. The S_2_ and S_3_ states are typically of ππ* character and carry much higher oscillator strength in the region 240–320 nm. Some species, however, are known to show exceptions to this state ordering, as will be discussed later.

Towards the end of the 1970s, the first laser-based picosecond transient absorption measurements in room-temperature solution were reported^25–30^. These studies typically involved excitation close to 355 nm and interrogated the flow of population out of the initially prepared S_1 _(nπ*) state via the competing ISC and IC pathways. Significant variation in the decay lifetime was observed, ranging from 22 ps in quinoxaline to 1580 ps in 1,6-naphthyridine. It was also found that quinoxaline displays a much greater propensity for ISC (*Φ*_ISC_ = 0.99) compared to quinazoline or 1,5-naphthyridine (*Φ*_ISC_ = 0.70 and 0.55, respectively) in isooctane solvent. There is, however, no clear correlation in these data between S_1_ state lifetime and ISC probability. Large ISC quantum yields in quinoxaline (*Φ*_ISC_ ≥ 0.90) were also observed upon 337.1 nm excitation in ethanol^31^ and benzene^32^ solutions, with the latter study reporting a much smaller value for isoquinoline (*Φ*_ISC_ = 0.21).

In recent years, several additional studies have appeared, reflecting an uptake of interest in the photochemistry of azanaphthalenes. This includes two comprehensive investigations into the gas-phase UV and VUV absorption spectroscopy of quinoline and isoquinoline^33,34^, and a theoretical coupled-cluster treatment of the low-lying excited states and ISC pathways in quinoxaline and quinazoline^35^. A time-dependent density functional theory study of IC vs ISC in quinoline and isoquinoline rationalised the differences between the molecules in terms of the energetic barrier for the IC pathway and the strength of the spin–orbit coupling (SOC) interaction^36^. Most recently, a resonance-enhanced multiphoton ionisation study combined with electronic structure calculations highlighted the role of the C–N–C angular coordinate in mediating vibronic coupling between the first two excited singlet states of isoquinoline^37^. That study also confirmed earlier predictions^34^ that the ordering of the two lowest-lying singlet excited states is inverted in this particular molecule, with ππ* below nπ* in the Franck–Condon region.

In the present work, we use ultrafast transient absorption spectroscopy (TAS) to study a carefully selected series of six azanaphthalenes in hexane solution. The molecules are excited at 267 nm and then interrogated using a broadband white-light continuum (WLC) probe spanning 340–750 nm. The measurements are coupled to a detailed theoretical mapping of the excited electronic states involved in the dynamics, allowing the experimental observations to be rationalised in a systematic manner across the entire series of related molecules.

## Results

**Fig. 2:**
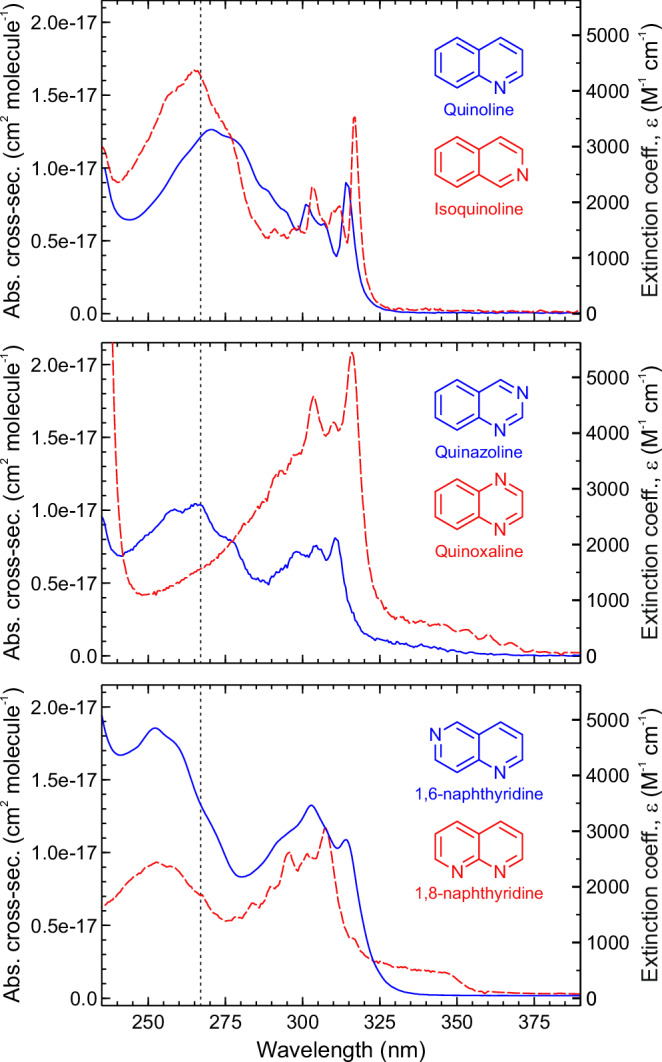
Room-temperature absorption cross-sections in hexane. (Top) quinoline (blue/solid), isoquinoline (red/dashed), (Middle) quinazoline (blue/solid), quinoxaline (red/dashed), (Bottom) 1,6-naphthyridine (blue/solid), 1,8-naphthyridine (red/dashed). Data were obtained using a simple Beer–Lambert law analysis (see section “Methods”). The vertical dashed lines indicate the central 267 nm pump wavelength used in our TAS measurements. No additional spectral features appear in the range 390–800 nm.

Room-temperature absorption spectra were obtained for all six molecules in advance of the TAS measurements. These data are presented in Fig. 2. In quinoline and isoquinoline, the magnitude of the cross-section is very similar to previously reported gas-phase measurements^33,34^. The positions of the various absorption band features are, however, red-shifted by ~5 nm in hexane (see Supplementary Fig. 1 for a direct gas-phase comparison in the case of quinoline). Most molecules studied here exhibit extremely similar absorption spectra across the wavelength region considered. The only partial exception is quinoxaline, but we note that the band structure of these data is very similar to that reported previously for the same system in vapour^38^. Theoretical calculations (see section “Methods”) confirm that 267 nm excites all molecules to a bright ππ* state. This corresponds to S_3_ in isoquinoline, quinoline, quinazoline and quinoxaline, and to S_4_ in 1,6- and 1,8-naphthyridine. We note that in quinoline, isoquinoline and 1,6-naphthyridine the character of S_1_ and S_2_ reverses in the Franck–Condon region, with ππ* sitting below nπ*. Supplementary Tables 1–12 provide a full list of calculated vertical excitation energies and associated oscillator strengths, including the dominant (vertical) character for each state.

**Fig. 3:**
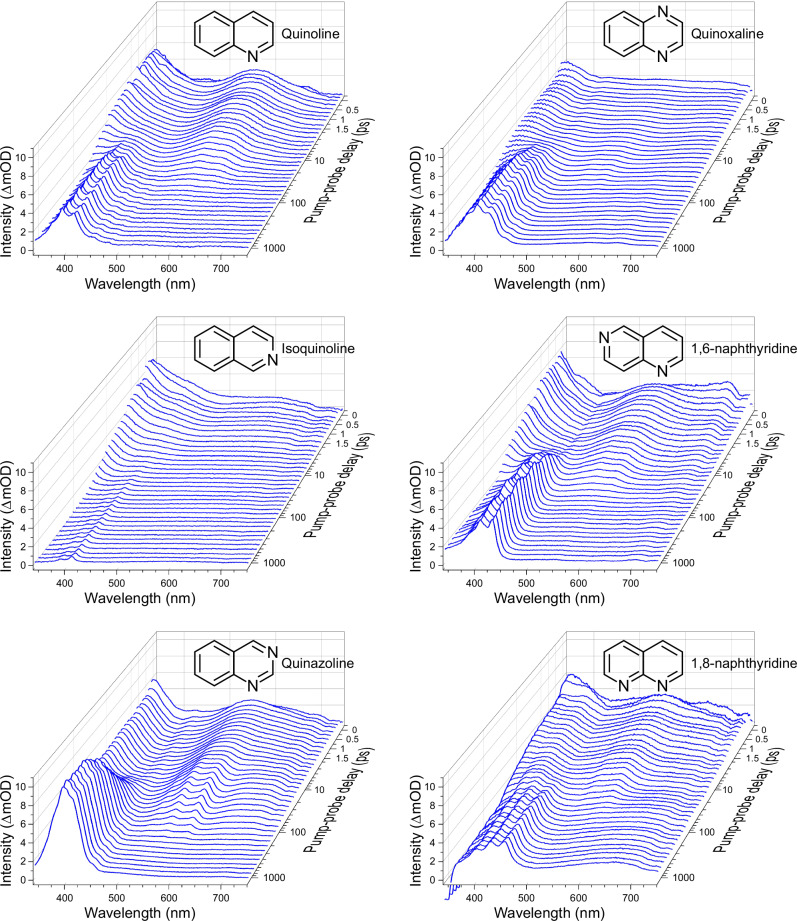
Chirp-corrected transient absorption spectra as a function of time (ps) and wavelength (nm) for all six molecules under study in hexane upon 267 nm excitation. Note the mixed linear-logarithmic scaling of the pump–probe time-delay axis. For clarity, the plots only include a subgroup of the complete pump–probe delay dataset (individual plotted spectra are spaced by 200 fs in the linear region and the logarithmic region shows every second time-delay sampled).

Figure 3 presents chirp-corrected time-resolved transient absorption spectra for all six molecules under study (see section “Methods” for details on data acquisition and processing). Each dataset exhibits two distinct peak features that originate at zero pump–probe delay and sit at the red (500–650 nm) and blue (<400 nm) regions of the probe spectrum. The relative amplitudes of these two features are broadly similar in all cases, and their decay matches the rise of a third, structured absorption band centred around 400 nm. This third band persists beyond the temporal observation window and exhibits a considerable variation in amplitude across the molecular series. It is over one order of magnitude larger in quinazoline than in isoquinoline, with the remainder of the molecules sitting between these two extremes.

Chirp-corrected TAS data are analysed using a Levenberg–Marquardt fitting routine which assumes the 2D dataset $S\left(\lambda ,\,\Delta t\right)$ may be expressed as a weighted sum of $n=3$ exponentially decaying functions,

1 $$S\left(\lambda ,{{\Delta }}t\right)=\mathop{\sum }\limits_{i=1}^{n}{A}_{i}(\lambda ) \cdot \exp [-\Delta t/{\tau }_{i}]\otimes g\left(\Delta t,\lambda \right)$$

**Fig. 4:**
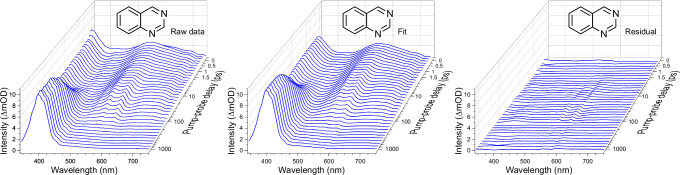
Chirp-corrected transient absorption spectra of quinazoline following 267 nm pump excitation. Raw data (left), a three-exponential function fit to these data using the procedure outlined in the text (centre), and the associated residual—i.e. the raw data minus the fit (right). Equivalent data for quinoxaline and 1,8-naphthyridine are presented in Supplementary Fig. 2. For clarity, the plots only show a subset of the complete pump–probe delay dataset (individual plotted spectra are spaced by 200 fs in the linear region and the logarithmic region where every second step is shown). All recorded timesteps beyond +250 fs are used in the fitting process.

For each exponential term, the aim is to obtain a set of wavelength-dependent amplitudes ${A}_{i}(\lambda )$ that constitute a decay-associated spectrum (DAS) attributable to a dynamical process with a specific lifetime *τ*_*i*_. These functions are convolved with the experimentally determined Gaussian cross-correlation function $g\left(\Delta t,\lambda \right)$, which varies approximately linearly between 170 and 130 fs FWHM over the 340–750 nm spectral bandwidth of the probe. An example of the fitting results is shown in Fig. 4 for the case of quinazoline (with additional illustrations for quinoxaline and 1,8-naphthyridine included in Supplementary Fig. 2). The associated residuals are small and show no sign of any systematic error, indicating that our fitting model is reliable and appropriate. One final detail to note is that the fitting procedure only considers data at pump–probe delays from +250 fs onwards to avoid the short-lived coherent artefact signal (CAS) that originates from the hexane solvent. This means, however, that any extremely fast (i.e. <300 fs) dynamics may be omitted from our model. To verify that this is not an issue, we re-evaluate our fitting process using data spanning the full range of pump–probe delay positions, with the *τ*_1–3_ values determined in the original (+250 fs onwards) analysis held fixed. When this is done, the residuals are almost identical to those of the independently recorded CAS signal (as shown in Supplementary Fig. 3). We can therefore be confident that our fitting strategy captures all relevant dynamical information within the spectral window of our probe. Table 1 provides a summary of all numerical time constants obtained.

**Table 1:** Summary of fitted lifetimes and associated 1σ uncertainties for all six molecules when excited by 267 nm in hexane solution

	*τ*_1_ (ps)	*τ*_2_ (ps)	*τ*_3_(ns)	S_3_ min	S_2_ min	Barrier to S_1_/S_0_ MECI (eV)	SOC at S_1_/T_2_ MECP (cm^−1^)
Isoquinoline	2.8 ± 0.1	22 ± 1	>50	No	No	0.06	0.02
Quinoline	2.8 ± 0.1	39 ± 1	>50	No	No	0.09	6.92
Quinazoline	11.2 ± 0.3	167 ± 1	>50	Yes	Yes	0.29 and 0.50	4.93
Quinoxaline	4.8 ± 0.4	57 ± 1	>50	No	No	0.51	8.68
1,6-naphthyridine	4.0 ± 0.3	37 ± 1	>50	No	No	0.1 and 0.16	3.47 and 9.43
1,8-naphthyridine	5.2 ± 0.3	61 ± 2	>50	No	No	0.25	6.31

Also included is a summary of the theoretically determined key parameters of the electronic structures which help rationalise the *τ*_1_ and *τ*_2_ lifetimes. This includes the presence of energy minima on the S_3_ and S_2_ electronic states, the energy barrier for accessing the S_1_/S_0_ minimum energy conical intersection (MECI) and the value of the spin–orbit coupling at the S_1_/T_2_ minimum energy crossing point (MECP). For further details see text.

**Fig. 5:**
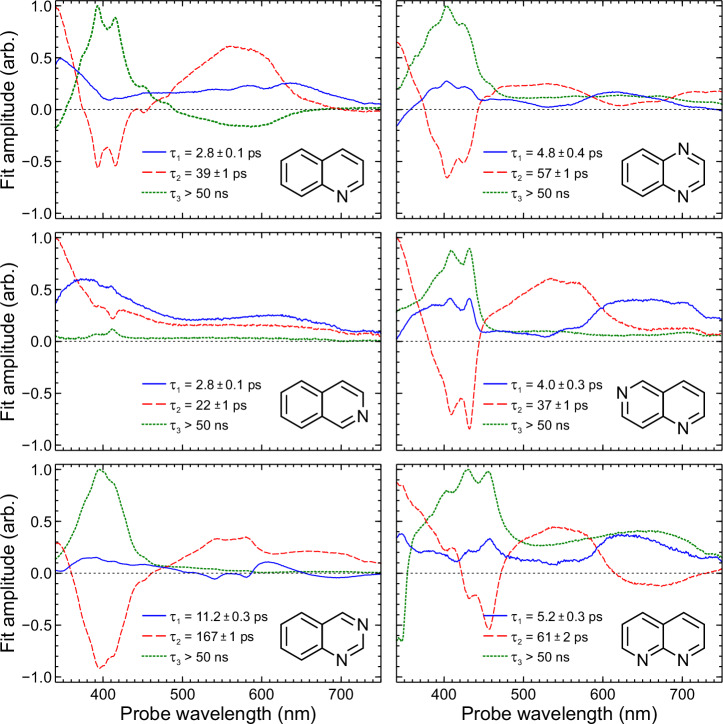
Decay-associated spectra (DAS) obtained for all six molecules under study. Plots are generated from a wavelength-dependent fitting analysis applied to the chirp-corrected TAS data (see Fig. 4). See the text for further details.

DAS plots for all six species under study are presented in Fig. 5. The presence of negative amplitude features is a direct consequence of the parallel fitting model described in Eq. (2), where all exponential decay functions have their origin at zero pump–probe delay. Negative DAS signals therefore indicate exponential growth in the excited-state population of an electronic state that is not populated by the initial pump step (i.e. they describe a spectral feature that arises through a sequential excited-state population transfer process—for example, via IC or ISC). This choice of a parallel basis for extracting kinetic information has the advantage of assuming no a priori information about the nature (sequential or otherwise) of the dynamical processes involved when fitting data. Some instructive illustrative examples of this effect (framed in the context of time-resolved photoelectron spectroscopy) may be found elsewhere^39^. Although there is no negative amplitude in the *τ*_2_ DAS of isoquinoline, there is nonetheless a shallow trough close to 405 nm. This trough is a consequence of the same sequential population transfer process as in the other systems under study but reflects a situation where the propensity to follow that pathway is considerably reduced (as also revealed in the much smaller amplitude of the corresponding *τ*_3_ DAS).

The *τ*_1_ DAS describes a process on the order of a few picoseconds that exhibits two broad bands sitting at the red (500–650 nm) and blue (<400 nm) regions of the probe spectrum. This signal is attributed to decay of the initially populated excited state following 267 nm absorption, which—as mentioned earlier and expanded upon later—is S_3_(ππ*) in all cases other than 1,6- and 1,8-naphthyridine, where S_4_(ππ*) becomes the relevant transition. The values obtained for *τ*_1_ in quinoline and isoquinoline are identical within experimental errors (2.8 ps) and are smaller than those seen in quinoxaline, 1,6-naphthyridine and 1,8-naphthyridine (4.0–5.2 ps). Of note, however, is the significantly larger *τ*_1_ obtained for quinazoline (11.2 ps). This outlying behaviour continues upon examining the *τ*_2_ DAS, where once again quinazoline displays a much larger time constant (167 ps) than any of the other molecules. Among the other systems considered there is, though, a greater variation compared to *τ*_1_, with *τ*_2_ values spanning a range between 22 ps (isoquinoline) and 61 ps (1,8-naphthyridine). In all six molecules, the shape of the negative amplitude in the *τ*_2_ DAS matches the positive feature in the *τ*_3_ DAS in the 350–450 nm region. This indicates a sequential process, as discussed earlier. Temporally, the feature described by the *τ*_3_ DAS persists well beyond the 1.4 ns observation window, and we therefore only quote a lower bound for this lifetime of 50 ns. The true value is known to greatly exceed this value, being on the order of microseconds^37,40^. The appearance of the *τ*_3_ DAS is very similar to spectra reported previously in triplet–triplet absorption measurements conducted on quinoline, isoquinoline and quinoxaline^19,22,41^. It is therefore straightforward to attribute the long-lived signals seen in our data to the lowest triplet state, which ultimately must be populated via ISC from the singlet manifold. Given the sequential link between the processes described by the *τ*_2_ and *τ*_3_ DAS, the *τ*_2_ lifetime may consequently be assigned to decay of the S_1_ state. The range of S_1_ lifetimes presented here are, in general, an order of magnitude faster than those obtained upon direct excitation at 355 nm (in cases where a direct comparison can be made)^30^. This is unsurprising given the significantly higher vibrational energy present when S_1_ is populated by IC from higher-lying electronic states excited at 267 nm. Of particular interest here, however, is the variation in relaxation timescales across this series of molecules and how this relates to apparent differences in ISC propensity.

## Discussion

**Fig. 6:**
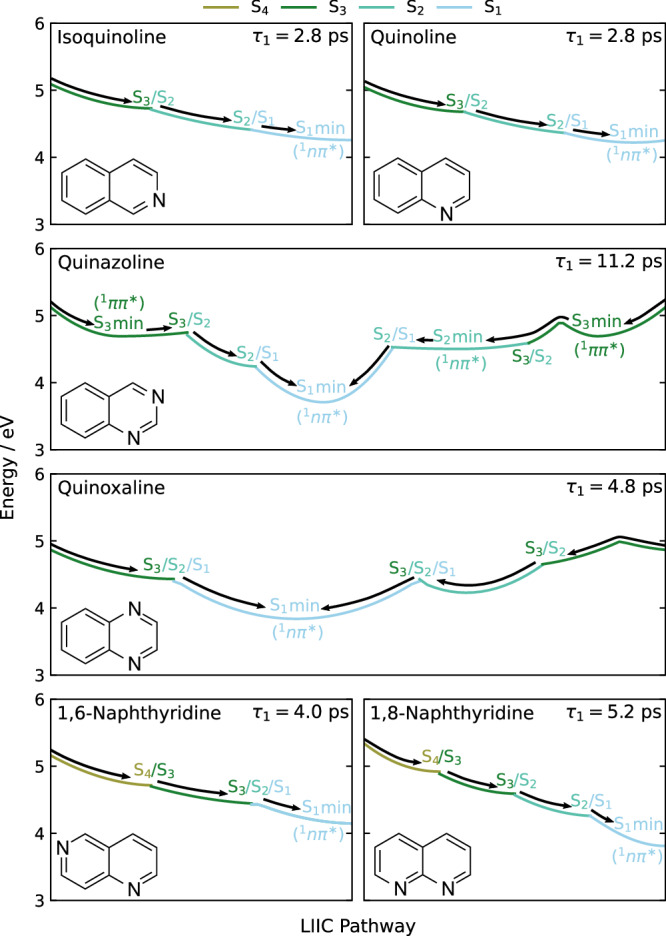
Potential energy profiles of the photorelaxation pathways from S_3_ (S_4_ for 1,6- and 1,8- naphthyridine) onto the S_1_ surface, for all six molecules, computed using SCS-ADC(2)/cc-pVDZ. The profiles start at the ground-state equilibrium geometry and are linearly interpolated between minimum energy conical intersections (MECIs) and excited-state minima. For clarity, we only show the active state involved along the pathway to the S_1_ (^1^nπ*) minimum (see Supplementary Fig. 5 for an additional version of this figure including all inactive states). We note that isoquinoline, quinoline and 1,6-naphthyridine have multiple S_1_ minima, all of which are accessible via the pathways shown here, however, only one is included for simplicity. These additional minima are discussed later in the text and are included in Fig. 7. The dominant electronic character of each excited-state minimum is denoted in brackets.

**Fig. 7:**
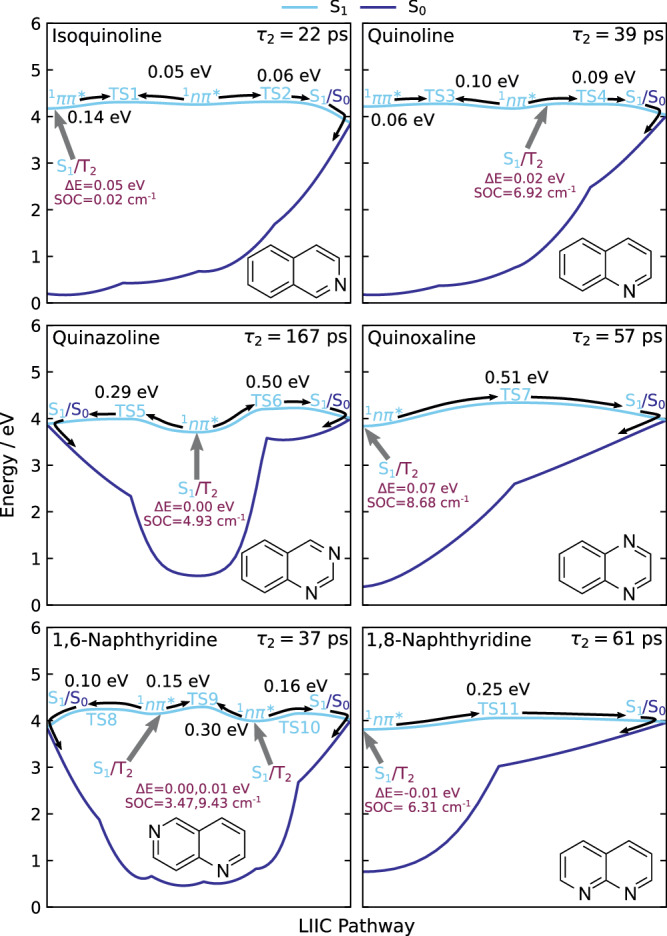
Potential energy profiles along the S_1_ surface leading to the S_1_/S_0_ MECIs, performed at the SCS-MP2/SCS-ADC(2)/cc-pVDZ level. These profiles have been computed using linear interpolation in internal coordinates (LIIC) between excited-state minima, the connecting transition states, and S_1_/S_0_ MECIs except between the S_1_ (^1^nπ*) minimum and TS5, TS6, TS10 and TS11, in quinazoline, 1,6-naphthyridine and 1,8-naphthyridine respectively, where minimum energy pathway (MEP) calculations were performed. The S_1_ minima are denoted by their dominant electronic character (^1^ππ* and ^1^nπ*). Additionally, the approximate locations of the S_1_/T_2_ MECPs are indicated by grey arrows, with their relative energies compared to the nearest S_1_ minimum and spin–orbit coupling (SOC) values given in purple.

In the following, we use quantum chemical calculations to rationalise the experimental observations, mapping them onto features of the excited electronic states such as minimum energy conical intersections (MECIs) and energy barriers, as well as minimum energy crossing points (MECPs) and associated SOCs. The calculations explain the systematic variation in singlet state lifetimes and the propensity for ISC observed experimentally. Table 1 includes a summary of the key features in the electronic structure that are linked to the *τ*_1_ and *τ*_2_ lifetimes observed in the molecules. To interpret the *τ*_1_ lifetimes, which correspond to IC decay from the initially excited state to the S_1_ state, we examine the potential energy surfaces for S_1–3_ (S_1–4_ in 1,6- and 1,8-naphthyridine) as shown in Fig. 6, using linear interpolation in internal coordinates (LIICs) between key points. Next, we examine the *τ*_2_ lifetimes, connected to the decay out of S_1_ via either IC or ISC. In Fig. 7, we show the LIICs that characterise the IC relaxation of S_1_. We also find that *τ*_1_ and *τ*_2_ relate to changes in local geometry at the N atoms in the molecules, as discussed further below. We conclude with an analysis of the triplet-state dynamics associated with the slow *τ*_3_ lifetimes.

### Decay to the S_1_ state (*τ*_1_)

**Fig. 8:**
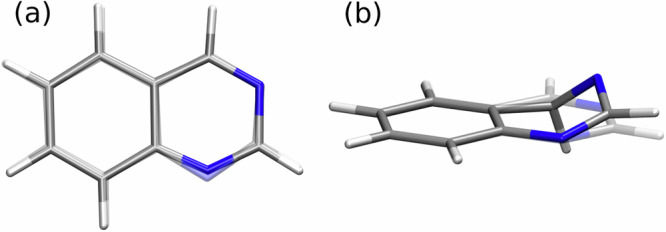
Key geometrical changes in quinazoline during the overall relaxation process. Firstly, **a** shows the changes in the C–N–C bond angles due to the nitrogen-centred ring in-plane bend, which occurs as the molecule decays to the S_1_ state from the initially excited ππ* state. Secondly, **b** displays the typical out-of-plane distortion, which occurs as the S_1_/S_0_ MECIs are approached from the S_1_ (^1^nπ*) minimum. Data are representative of behaviour seen in all six systems under study.

The most rapid decay from the initially excited state to the S_1_ electronic state occurs in quinoline and isoquinoline, with a *τ*_1_ = 2.8 ps decay constant that is unaffected by the position of the single N centre. The LIICs in Fig. 6 show that both these molecules form an effective funnel towards S_1_ via two consecutive MECIs (S_3_/S_2_ and S_2_/S_1_) which coincide with minima on the potential energy surfaces. This leads to a barrierless cascade from the initially excited S_3_ state to the S_1_ surface and the S_1_ (^1^nπ*) minimum. The passage through the MECIs is associated with changes in the C–N–C bond angles that preserve the planarity of the rings. This observation holds true for all six molecules and is illustrated for quinazoline in Fig. 8.

Adding a second N atom to the molecule slows down the dynamics. We first consider 1,6-naphthyridine and 1,8-naphthyridine, which both have one N atom per ring. These molecules are excited to the S_4_ bright state, rather than S_3_, but otherwise resemble quinoline and isoquinoline, forming a barrierless funnel to the S_1_ state as shown in Fig. 6. The resulting IC cascade is more efficient in 1,6-naphthyridine than in 1,8-naphthyridine since only two MECIs are involved, first S_4_/S_3_ and then a three-state S_3_/S_2_/S_1_. This involves smaller distortions of the molecular geometry and yields a shorter *τ*_1_ lifetime at 4 ps. In 1,8-naphthyridine, the barrierless pathway instead involves three consecutive conical intersections, S_4_/S_3_, S_3_/S_2_, and finally S_2_/S_1_. This increases the *τ*_1_ lifetime to 5.2 ps, with quite significant expansion in the C–N–C bond angles required to reach the S_3_/S_2_ MECI from the S_4_/S_3_ MECI, and then a reversal of the geometry changes required to find the S_2_/S_1_ MECI (which is located close to the S_4_/S_3_ MECI).

Finally, we examine the pair of molecules quinoxaline and quinazoline with the two N centres located on a single ring. Compared to quinoline and isoquinoline, the dynamical timescales are significantly slower, and the topography of the excited electronic state potential energy surfaces is complicated, with two or more distinct decay pathways emerging in both molecules. Quinazoline has the largest *τ*_1_ lifetime out of all six molecules at 11.2 ps, while quinoxaline is closer to 1,6-naphthyridine and 1,8-naphthyridine with *τ*_1_ = 4.8 ps. This may seem surprising given the complex pathway shown for quinoxaline on the right-hand side of the panel in Fig. 6, which proceeds over a small 0.1 eV barrier (noting that the barrier height is an upper bound given that the pathway is constructed by linear interpolation) and through a S_3_/S_2_ MECI, followed by passage through a three-state S_3_/S_2_/S_1_ MECI accessed through a relatively large change in geometry. However, the second pathway, shown on the left-hand side of the same panel, directly accesses the three-state S_3_/S_2_/S_1_ MECI. This opens a more immediate route to the S_1_ state, reminiscent of the funnels in 1,6- and 1,8-naphthyridine and explains the faster dynamics in quinoxaline vs quinazoline. Although we do not explicitly consider LIIC pathways starting from S_2_—which would be the state accessed predominantly at longer excitation wavelengths (in the approximate region 285–325 nm)—we note that if one was to consider starting from this state, population would pass through the same three-state S_3_/S_2_/S_1_ MECI (see Supplementary Fig. 5).

The slow dynamics in quinazoline deserve the most detailed discussion. It may be attributed to the presence of distinct minima separated by small barriers on both the S_3_ and S_2_ potential energy surfaces. This stands in contrast to the other five molecules, where the potential minima coincide with MECIs. Figure 6 shows the two main relaxation channels in quinazoline upon excitation to the S_3_ state. The first channel, starting on the left-hand side, passes through the S_3_ minimum and undergoes IC through a S_3_/S_2_ MECI onto the S_2_ surface. From here, a barrierless transition onto S_1_ through an S_2_/S_1_ MECI bypasses the S_2_ minimum. The second pathway, on the right-hand side of the panel, passes through the same S_3_ minimum and then over a 0.2 eV energy barrier (noting, as before for quinoxaline, that the barrier height is an upper bound given that the pathway is constructed by linear interpolation). The molecule then reaches the S_2_ minimum via a second S_3_/S_2_ MECI. The minimum is located near the S_2_/S_1_ MECI which allows for subsequent decay to the S_1_ state. Additionally, a third pathway is present (shown in Supplementary Fig. 6). This is accessed via a transition state (TS) located 0.03 eV above the S_2_ minimum and proceeds through the same S_2_/S_1_ MECI as the pathway on the left-hand side of Fig. 6. Overall, the quinazoline IC topography is rather corrugated and the dynamics involves (at least) two distinct conical intersections, several potential energy minima, and (in two out of three pathways) potential energy barriers. These factors all contribute to the notably slower *τ*_1_ dynamics in quinazoline.

### Relaxation of the S_1_ state (*τ*_2_)

The trends in the *τ*_2_ lifetimes are explored by examining the features on the S_1_ potential energy surfaces. For IC, we locate minima, transition states (TSs), and S_1_/S_0_ MECIs on the S_1_ potential energy surfaces for all six molecules. The relevant LIICs are shown in Fig. 7. The LIICs are calculated between minima, TSs, and MECIs, except between the S_1_ minimum and the TS5, TS6, TS10 and TS11 TSs in quinazoline, 1,6-naphthyridine and 1,8-naphthyridine, where minimum energy pathway (MEP) calculations are performed instead, since the LIICs yield artificial maxima before the true TSs in those cases. From our calculations, we identify that all singlet photorelaxation pathways to the ground-state proceed via the S_1_ (^1^nπ*) minimum. Interestingly, the motion on the S_1_ surface corresponds to the same nitrogen-centred ring in-plane bend that accompanies decay from S_3_ (and S_4_) to the S_1_ surface, but with additional distortion out of the plane of the aromatic rings when approaching the S_1_/S_0_ MECIs. These out-of-plane distortions, shown in Fig. 8 for quinazoline, are likely to be responsible for *τ*_2_ being significantly slower than *τ*_1_.

Competing with the IC is ISC to the triplet-state manifold. The propensity for ISC is investigated by locating MECPs between singlet and triplet states and by calculating the SOC at these geometries. From our computations, ISC is predicted to occur via an S_1_/T_2_ MECP for all six molecules. The MECPs are accessed without the molecules having to cross any barriers. We thus attribute the *τ*_2_ lifetimes to a combination of the potential energy barriers for reaching the S_1_/S_0_ MECIs and the SOC at the S_1_/T_2_ MECPs, which are summarised in Table 1.

As seen in Table 1 and Fig. 3, isoquinoline has the shortest *τ*_2_ lifetime (22 ps) and displays a very small propensity to undergo ISC into the triplet state—as also indicated by the small *τ*_3_ DAS amplitude in Fig. 5. The S_1_/T_2_ MECP is easily accessible from the ^1^ππ* minimum, but the SOC has a magnitude of only 0.02 cm^−1^ due to both the S_1_ and T_2_ states having dominant ππ* character at this geometry. Given the absence of ISC and the known lack of fluorescence in this system, the decay of the S_1_ state is therefore predominantly via ground-state re-population by IC. Data presented in Fig. 7 indicate that isoquinoline has two S_1_ minima, which are likely to be populated equally after IC onto the S_1_ surface. An energy barrier of 0.14 eV from the ^1^ππ* minimum must be overcome to access the ^1^nπ* minimum, while the reverse process has a barrier of 0.05 eV. Once in the ^1^nπ* minimum, there is a small barrier of 0.06 eV to access the S_1_/S_0_ MECI. Since the heights of both barriers from the ^1^nπ* minimum are comparable, ground-state relaxation and exploration of the S_1_ surface are likely to compete, slowing down the dynamics.

Quinoline and 1,6-naphthyrdine have similar *τ*_2_ lifetimes of 39 and 37 ps, respectively. As in isoquinoline, two minima exist on the S_1_ surface in quinoline, and there is one accessible S_1_/S_0_ MECI from the ^1^nπ* minimum. From this minimum, there is a barrier of 0.09 eV to access the MECI in addition to a barrier of 0.10 eV leading to the ^1^ππ* minimum (see Fig. 7). The S_1_/T_2_ MECP lies 0.02 eV above the ^1^nπ* minimum and has a SOC magnitude of 6.92 cm^−1^. At this geometry, the S_1_ state has nπ* character and T_2_ has mixed ππ*/nπ* character, giving rise to a sizeable SOC effect. The differences in the energy barriers to access the ground state and in the SOC values rationalise the increase in *τ*_2_ lifetime compared to isoquinoline. We note that the S_1_/T_2_ MECP in quinoline lies along the pathway to the easily accessible S_1_/S_0_ MECI, and hence, population transfer to the ground state may affect the amount of ISC that occurs.

In contrast to isoquinoline and quinoline, three minima are located on the S_1_ surface in 1,6-naphthyridine, one ^1^ππ* minimum and two ^1^nπ* minima. The latter connects to the S_1_/S_0_ MECIs, which both exhibit out-of-plane distortions. For clarity, only the two ^1^nπ* minima are shown in Fig. 7. From the ^1^nπ* minimum with an increased C_5_–N_6_–C_7_ bond angle, there is a barrier of 0.10 eV via TS8 to access the respective S_1_/S_0_ MECI, which is akin to the barrier in quinoline. In addition, from the ^1^nπ* minimum with an increased C_8a_–N_1_–C_2_ bond angle, there is a barrier of 0.16 eV to the S_1_/S_0_ MECI, via TS10. Interconversion between the two ^1^nπ* minima is unlikely as the barrier between them is larger from each direction than the barriers to the MECIs. For ISC, we found that the S_1_/T_2_ MECP near the ^1^nπ* minimum with a larger barrier to the S_1_/S_0_ MECI has a large SOC value of 9.43 cm^−1^ due to the S_1_ state having nπ* and the T_2_ ππ* character, likely leading to preferential ISC into the triplet manifold over relaxation to the ground state. A second S_1_/T_2_ MECP with a smaller SOC value of 3.47 cm^−1^ is located near the ^1^nπ* minimum with a smaller barrier to the S_1_/S_0_ MECI. Hence, at this S_1_ minimum, ground-state relaxation is favourable over ISC.

Quinoxaline has a *τ*_2_ lifetime of 57 ps, which is similar to that of 1,8-naphthyridine (61 ps) despite having a significantly larger energy barrier of 0.51 eV from the S_1_ (^1^nπ*) minimum to the only accessible S_1_/S_0_ MECI. However, the S_1_/T_2_ MECP lies only 0.07 eV above the S_1_ (^1^nπ*) minimum and has a large SOC value of 8.68 cm^−1^, attributed to a change in character from S_1_ (nπ*) to T_2_ (ππ*). Thus, we propose that ISC into the triplet manifold is likely dominant over relaxation to the ground state, which is in broad agreement with previous reports at longer excitation wavelengths^30–32^.

The *τ*_2_ lifetime in 1,8-naphthyridine of 61 ps is attributed to a combination of factors. Firstly, from the S_1_ (^1^nπ*) minimum, there is a barrier of 0.25 eV to the only accessible S_1_/S_0_ MECI. As discussed above, the impact of this lower barrier height compared to quinoxaline is compensated by the stronger SOC in quinoxaline. The S_1_/T_2_ MECP located at the S_1_ (^1^nπ*) minimum in 1,8-naphthyridine has a SOC value of 6.31 cm^−1^, again assigned to a change in character from S_1_ (nπ*) to T_2_ (ππ*). This is similar to that of quinoline, which has a faster S_1_ decay (*τ*_2_ = 39 ps) but we recall that 1,8-naphthyridine has a significantly larger TS barrier than quinoline, slowing down the rate of IC to the ground state.

Finally, quinazoline displays by far the longest *τ*_2_ lifetime (167 ps) amongst all the systems considered in the present study. This is due to a combination of factors. Although the molecule has two S_1_/S_0_ MECIs accessible from the S_1_ (^1^nπ*) minimum, both are associated with large energy barriers. The first involves distortion of the molecular geometry at the N_1_ and C_2_ atoms and has a barrier of 0.50 eV. The second involves distortion at the N_3_ and C_4_ atoms with a barrier of 0.29 eV, which is larger than the barriers for all molecules except quinoxaline. At the same time, the SOC value at the S_1_/T_2_ MECP is 4.93 cm^−1^, which is significantly smaller than both 1,8-naphthyridine (6.31 cm^−1^) and quinoxaline (8.68 cm^−1^) despite S_1_ having dominant nπ* and T_2_ having dominant ππ* character at this geometry. The result is a slow rate of ISC, in agreement with previous literature findings^30^. The net outcome of the slow rates of ISC and IC is the significantly longer lifetime in the S_1_ state.

### Dynamics in the triplet state (*τ*_3_)

The time constant *τ*_3_ is nominally ascribed to the lifetime of the T_1_ state, however, our calculations show that the triplet manifold is entered via an S_1_/T_2_ MECP. A single time constant for the triplet manifold exists because the molecules transition rapidly through the T_2_ state onto the T_1_ surface. We find that only isoquinoline, quinoline and quinoxaline have T_2_ minima, with ππ* character in isoquinoline and nπ* character in quinoline and quinoxaline. In quinoline and quinoxaline, the potential energy surfaces from the S_1_/T_2_ MECP to the T_2_/T_1_ MECI are rather flat, with only minimal changes to the molecular geometry necessary to access the T_2_/T_1_ MECI and no discernible impact from the presence of the minima. We therefore expect them to transition rapidly to the T_1_ state. In isoquinoline, the T_2_/T_1_ MECI is located at higher energy than the S_1_/T_2_ MECP, however, the molecule is unlikely to enter the triplet manifold at this geometry given the very small SOC values dictated by the symmetry of the S_1_ and T_2_ electronic states close to the Franck–Condon region. Moreover, in quinazoline, 1,6- and 1,8-naphthyridine the T_2_ minimum coincides with the T_2_/T_1_ MECI and hence, these systems are likely to move rapidly to T_1_. A full set of relevant LIICs and MECPs is given in Supplementary Figs. 7 and 8.

All six molecules contain one T_1_ minimum, which has ππ* character and hence, they all produce the characteristic signal in the TAS spectrum around 400 nm. There is a barrierless transition from each T_2_/T_1_ MECI to the T_1_ minimum. Consequently, once the T_2_/T_1_ MECI is accessed, each molecule will directly enter the T_1_ minimum and is expected to remain in this minimum for longer than 50 ns.

## Conclusions

Our findings demonstrate the ability of ultrafast transient absorption to yield rich information on complex photoexcited dynamics in solution, mapping reaction coordinates over broad time- and spectral-ranges. Using quantum chemical calculations, we can rationalise the dynamics in terms of simple features such as MECIs, SOC at MECPs, and energy barriers. This allows us to discover important trends and correlations in the photophysics of a sequence of molecules formed by substituting nitrogen atom(s) into varying positions in naphthalene. Although the molecules in this sequence are closely related, they exhibit a remarkable variation in their photophysics.

The experiments identify three distinct timescales in the photoexcited molecules: (1) rapid IC to the first excited singlet state (*τ*_1_), (2) competition between IC to the singlet ground state and ISC to the triplet manifold (*τ*_2_), and (3) slow evolution on the lowest triplet state (*τ*_3_). The first timescale (*τ*_1_) is characterised by a cascade through a sequence of conical intersections, especially efficient in the two molecules with only one nitrogen atom (quinoline and isoquinoline). Adding a second N atom, particularly to the same ring, slows down the progression by complicating the topography of the excited-state potential energy surfaces and restricting access to the MECIs. Across all molecules, *τ*_1_ correlates closely with the number of MECIs en route to S_1_ and whether minima or potential barriers are present. The second timescale (*τ*_2_) is characterised by the competition between S_1_ to S_0_ IC and ISC to T_2_. Here, the speed of IC relates closely to the height of the potential barriers, which are lowest for quinoline and isoquinoline, higher for 1,6- and 1,8-naphthyridine (one nitrogen atom per ring), and highest for quinoxaline and quinazoline (two nitrogen atoms in the same ring). Notably, we find that the changes in molecular geometry around the N atoms may play a key role in setting the overall *τ*_2_ timescales, with the slower rate of IC attributable to out-of-plane distortions at the N centres. Finally, the strength of ISC is closely related to the SOC at the S_1_/T_2_ MECP, with all target molecules then decaying rapidly to the T_1_ state once they enter the triplet manifold.

Several natural extensions of this study can be envisioned. It would be interesting to expand the set of molecules, to examine the role of the excitation energy, and to investigate the effect of changing the solvent. From a theoretical point of view, it would be informative to undertake simulations to determine more closely how the characteristic features identified here relate to the actual dynamics. The type of investigations that this work represents provides the foundation for optimisation of photophysical properties informed by quantum chemical calculations and experiments, and, eventually, the intelligent design of molecules with specifically tailored photochemical properties.

## Methods

### Experimental

Preliminary room-temperature UV absorption spectra were recorded using a benchtop spectrophotometer (Shimadzu UV-2550). To obtain absolute cross-section information, data for each molecule were recorded in an absorption cell of length 1 mm at 11 different concentrations (0.8–2.0 mM) in hexane. The linear Beer–Lambert law relationship between the concentration and the natural logarithm of the transmitted intensity fraction then permitted extraction of the cross-section using a simple fitting procedure to obtain the gradient at each wavelength.

Time-resolved experiments were undertaken using a bespoke transient absorption spectrometer that has recently been described in detail elsewhere^42^. Briefly, a 200 mW portion of the 800 nm fundamental output from a 1 kHz Ti:Sapphire oscillator/regenerative amplifier combination (Spectra-Physics Tsunami/Spitfire Pro) was initially split in a 95:5 ratio to provide the starting input for the pump and probe beamlines, respectively. The pump beam (initially *Ø* = 1 cm) was reduced in diameter by around 70% using a simple Galilean lens telescope and then underwent frequency conversion to the third harmonic (267 nm) using a pair of thin β-barium borate crystals. For the present experiments, this UV beam was attenuated to an energy of 0.2 μJ pulse^−1^. In the probe arm, the 800 nm pulses were initially directed onto a gold corner-cube retroreflector mounted on a motorised linear translation stage. A λ/2 waveplate was then used to position the linear probe polarisation at the magic angle (54.7°) with respect to that of the pump, eliminating any time-dependent variation in absorption due to molecular axis alignment effects. Subsequent focusing through a fused silica lens (*f* = 5 cm) into a 1 mm CaF_2_ window induces self-phase modulation and produces a WLC spanning the visible and near-UV spectral region (340–750 nm). This beam was then recollimated using a curved aluminium mirror (*f* = 10 cm). The CaF_2_ window was set in continuous up-down translational motion to prevent optical damage (at a scan rate of 0.02 mm s^−1^).

Pump and probe beams were then focused independently using a pair of concave aluminium mirrors (*f* = 15 cm and *f* = 10 cm, respectively) and overlapped spatially with an intersection angle of ~6° inside a liquid flow cell (DLC-S25, Harrick Scientific Products).

All six molecules under study were purchased from TCI or Sigma-Aldrich and had a stated purity of 97% or higher. Solutions were prepared in hexane (≥95%, Fisher Scientific) at concentrations of ~20 mM—a value based on preliminary absorption spectroscopy data to ensure a linear absorption regime for the pump along the entire path length of the interaction region. Test measurements made using sample concentrations reduced by a factor of two and four reveal no appreciable change in the spectral or temporal response, indicating aggregation effects are not a factor (also see Supplementary Fig. 4). Circulation of room-temperature liquid samples between a reservoir vessel and the cell was achieved using a small peristaltic pump (Kamoer KCP-D) operating at a flow rate of 50 mL min^−1^. At the point of laser interaction inside the cell, the sample of interest passed between a pair of circular CaF_2_ windows (2 mm thick, 25 mm diameter) separated by a 100 μm thick PTFE spacer. The cell was mounted on a linear piezo motor stage which underwent side-to-side translation at a rate of 0.02 mm s^−1^. This eliminated issues with photoproduct deposition on the windows during periods of extended data collection. Every 30 min the cell was also translated manually in the vertical direction.

Following interaction with the sample, the WLC probe was recollimated using a fused silica lens (*f* = 10 cm) and then passed through a filter to remove the intense 800 nm portion of the spectrum. The remaining spectral profile (340–750 nm) was then analysed by a 1 kHz compact spectrometer (AvaSpec-ULS2048CL-EVO-RS-UA, Avantes) after coupling into an optical fibre. Shot-to-shot acquisition of white light spectra in the presence and absence of the pump pulse was achieved through the inclusion of a 500 Hz chopper wheel in the pump arm. A single scan of the temporal delay Δ*t* between the pump and the probe interrogated the transient dynamics between −500 and 1500 fs in 50 fs increments, with a further 60 exponentially increasing steps then extending out to 1.4 ns. The order of the individual timesteps was randomly sampled, and in each instance a total of 3000 single-shot white light spectra (1500 pump-on, 1500 pump-off) were collected. This overall process was then typically repeated for 15–20 scans and the accumulated intensity of the pump-on and pump-off white light spectra were directly compared to provide a measure of the molecular absorbance following UV irradiation. This is expressed as differential change in optical density (ΔmOD) for each timestep,

2 $${\Delta {\rm{mOD}}\left(\lambda ,\Delta t\right)=\log }_{10}\left(\frac{{I\left(\lambda \right)}_{{off}}}{{I\left(\lambda ,t\right)}_{{on}}}\right)\times 1000$$

Spectra collected over a range of different delay increments Δ*t* display a non-linear shift in the point of maximum temporal pump–probe overlap that increases towards longer wavelengths λ within the WLC. This is a consequence of chirping induced by transmission through optically dense media, and correction for this effect was applied prior to the extraction of any dynamical information. This was performed using the CAS obtained from a TAS measurement conducted on the pure hexane solvent under the same experimental conditions. A more expanded discussion of this procedure may be found in our earlier publication^42^.

When considering the model for the transient absorption spectra described by Eq. (1), individual data (i.e. wavelength) channels were initially fitted independently by three exponential functions to obtain a series of wavelength-dependent decay constants and associated amplitudes. The decay constants were then averaged across all data channels to give final (i.e. global) lifetimes *τ*_1−3_. A second fitting step was then conducted across all data channels to re-evaluate the ${A}_{i}(\lambda )$ DAS amplitudes using the (now fixed) global *τ*_1−3_. Given the large number of individual data channels, this approach is significantly less time-consuming and more robust than conducting a fully global (i.e. simultaneous time, wavelength and amplitude) fit.

### Theory

The ground-state equilibrium geometries of all six molecules were optimised using SCS-MP2 and the excited-state minima were optimised using SCS-ADC(2)^43,44^ using the cc-pVDZ basis set throughout (see Supplementary Data 1). The calculations were performed in Turbomole^45^, which was interfaced with CIOpt^46^ to locate MECIs and MECPs. TSs between excited-state minima were identified by firstly computing an MEP between the two minima of interest. The highest-energy geometry was then used as an initial guess for the optimisation of the TS. The optimised TS structure was confirmed by a vibrational frequency calculation, checking that the TS had one imaginary frequency. Finally, an intrinsic reaction coordinate calculation was performed to validate that the optimised TS connected the correct two minima. Potential energy profiles were constructed by LIICs between the relevant points along the potential energy surfaces. SOC matrix elements were computed at the same SCS-ADC(2)/cc-pVDZ level.

All excited states up to S_3_ are considered for isoquinoline, quinoline, quinazoline and quinoxaline. In these molecules, S_3_ is the bright ππ* state predominantly populated upon 267 nm (4.64 eV) absorption according to our vertical excitation energies and previous experiments^24,33,34^. For 1,6- and 1,8- naphthyridine, S_4_ must also be included, since this becomes the bright ππ* state. We also note that in quinoline, isoquinoline and 1,6-naphthyridine the character of S_1_ and S_2_ is reversed, with ππ* sitting below nπ*. A full list of calculated vertical excitation energies and associated oscillator strengths is provided in Supplementary Tables 1–12, including the dominant character for each state in the Franck–Condon region.

## Supplementary information

Supplementary Material

Description of Additional Supplementary Files

Supplementary Data 1

## Data Availability

Data are available from the corresponding authors upon reasonable request. See also Supplementary Data 1.
