# Supplementary material for: Excited state dynamics of azanaphthalenes reveal opportunities for the rational design of photoactive molecules
Source: Commun Chem. 2025 Jan 9;8:7. doi: 10.1038/s42004-024-01403-z (PMC11717923; doi:10.1038/s42004-024-01403-z)
Supplement: Supplementary file 1 — Supplementary Material [file 42004_2024_1403_MOESM1_ESM.pdf]

Excited state dynamics of azanaphthalenes reveal opportunities for the rational design of photoactive molecules

Malcolm Garrow, Lauren Bertram, Abi Winter, Andrew W. Prentice, Stuart W. Crane, Paul D. Lane, Stuart J. Greaves, Martin J. Paterson, Adam Kirrander and Dave Townsend

Supplementary Information

Additional transient absorption data and supporting analysis plus further quantum chemistry calculations.

1. Additional Experimental Figures

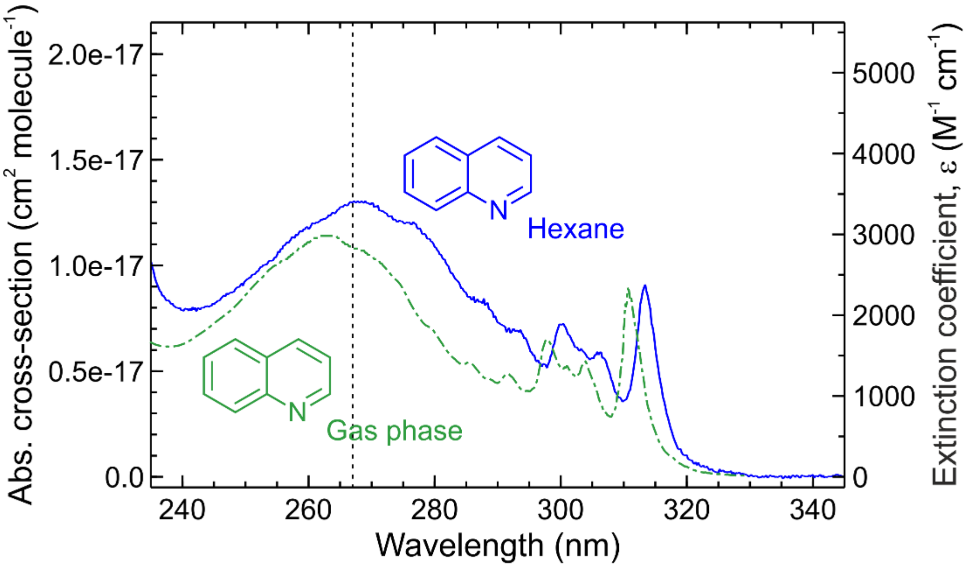

Supplementary Figure 1: Comparison of the quinoline UV absorption spectrum in hexane (solid blue line) and in the gas phase (dot-dash green line). The vertical dashed line indicates the central 267 nm pump wavelength used in our TAS measurements. The hexane data is reproduced from Fig. 2 in the main manuscript, and the gas phase data – originally reported by Leach *et al.* [*J. Phys. Chem. A,* **122** 5832 (2018)] – was obtained from the MPI-Mainz UV/VIS Spectral Atlas [*Earth Syst. Sci. Data* **5**, 365 (2013)].

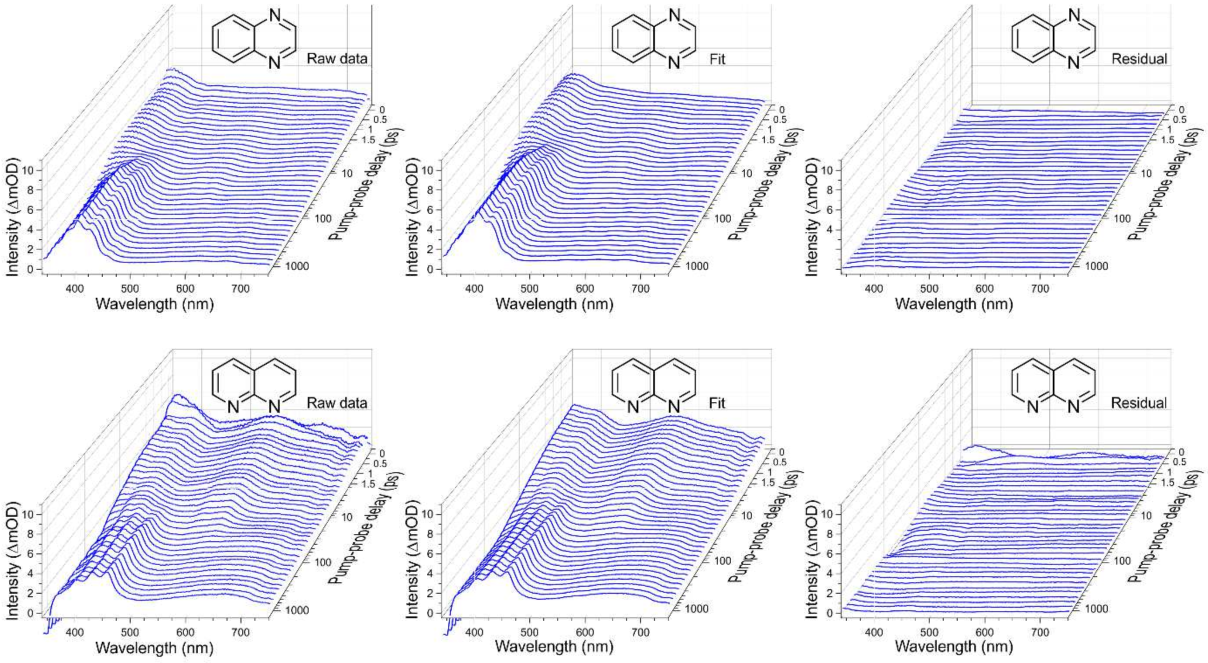

Supplementary Figure 2: Raw transient absorption data obtained following 267 nm excitation in quinoxaline (top row, left panel) and 1,8-naphthyridine (bottom row, left panel). Also included are three-exponential fits to this data – as detailed in the main manuscript text (middle panels), and the associated residuals (right panels). The 1,8-naphthyridine data shows a small ground state bleach that gives rise to negative intensity signals at probe wavelengths <350 nm – something that is absent in the other systems investigated in this work. For clarity, the plots only show a subset of the complete pump-probe delay dataset (individual plotted spectra are spaced by 200 fs in the linear region and every second step is shown in the logarithmic region). All recorded timesteps beyond +250 fs are used in the fitting process.

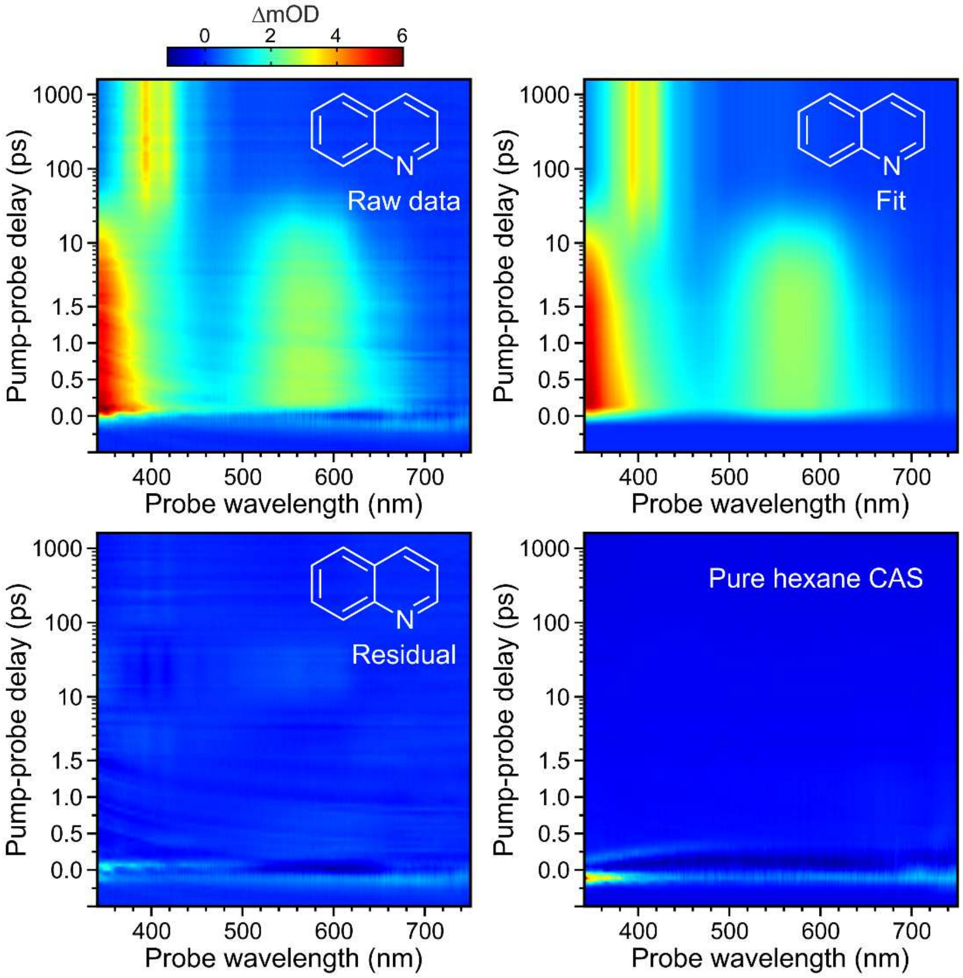

Supplementary Figure 3: An alternative, top-down view of the raw (chirp-corrected) TAS data obtained for quinoline following 267 nm excitation in hexane (*cf*. Fig. 3 in main text). Also included is a fit to this data over the full range of pump-probe delays using fixed time constants obtained when previously fitting from +250 fs onwards (see main text for further details). The residual (i.e. the raw data minus the fit) bears a very strong resemblance to the (chip-corrected) coherent artefact signal (CAS) recorded from the pure hexane solvent, indicating that there are no dynamical processes operating over the first few hundred femtoseconds in quinoline itself. Similar residuals are obtained for all other azanaphthalene species under study.

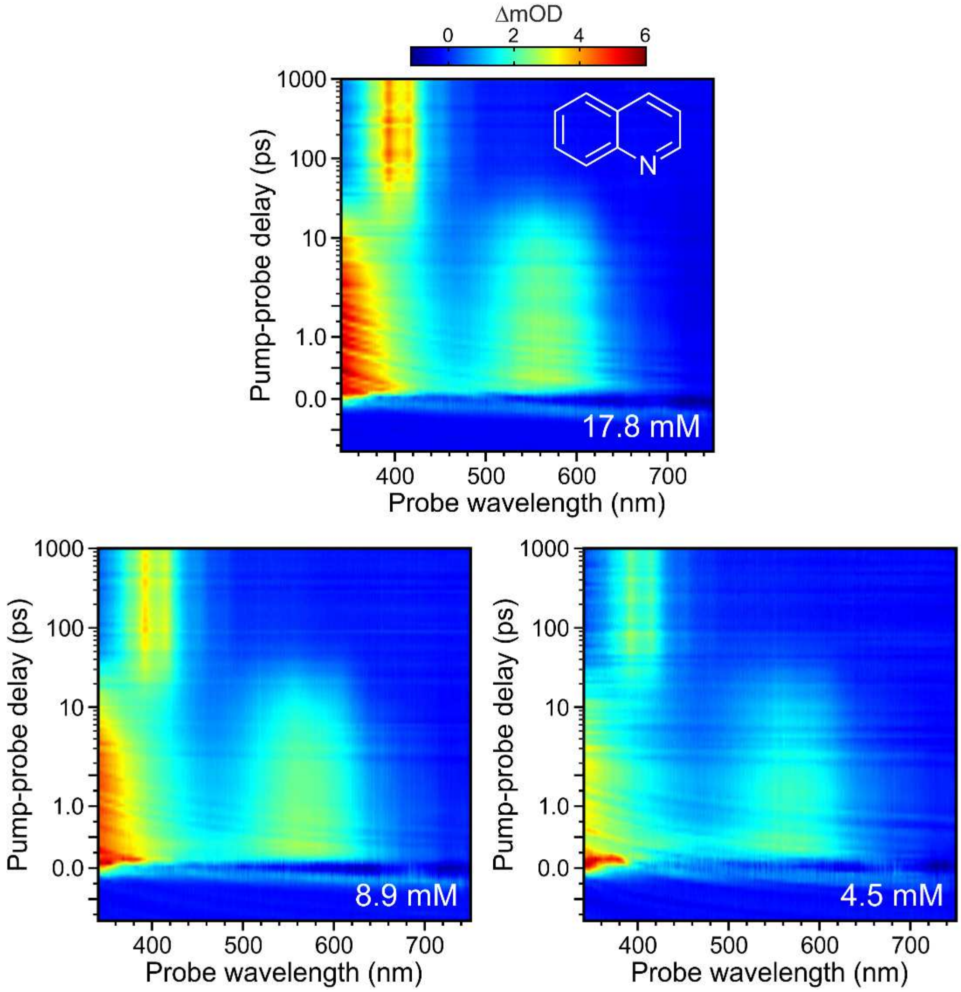

Supplementary Figure 4: Top-down view of the raw (chirp-corrected) TAS data obtained for quinoline following 267 nm excitation in hexane at three different sample concentrations (17.8 mM, 8.9 mM and 4.5 mM). In all cases the data reveal very similar spectral and temporal responses, indicating no significant aggregation effects.

2. Additional Theory Information

2.1 Vertical excitation energies

Vertical excitation energies were computed with SCS-ADC(2)/aug-cc-pVQZ from the ground-state equilibrium geometry optimized at the SCS-MP2/cc-pVDZ level of theory for all six molecules. Excitation energies are overestimated using the cc-pVDZ basis, and therefore, we have used a larger basis set to obtain sufficiently accurate energies, to confirm which state is excited at 267 nm. We show excitation energies, electronic state characters and oscillator strengths for the first three excited states (S_{1-3}) for isoquinoline, quinoline, quinazoline and quinoxaline as S_3 is the optically bright ππ* state, which is excited at 267 nm, whereas in 1,6-naphthyridine and 1,8-naphthyridine, S_4 is the relevant transition and hence, is also included for these two molecules. We note that the electronic states retain the same character between basis sets. Moreover, the excitation energies have been computed in the gas-phase and using the conductor-like screening model (COSMO) as an implicit solvation model of hexane. As shown below in Supplementary Tables 1-12, there are minimal differences between the gas phase and COSMO vertical excitation energies. Additionally, for all molecules except 1,8-naphthyridine there is a retention in the state character between the two methods. In 1,8-naphthyridine, the S_1 and S_2 states are only separated by an energy difference of 0.02 eV in the gas phase and are found to switch order when including solvent effects but remain very close in energy (0.06 eV). However, across the FC to S_4/S_3 MECI LIIC surface the character of the S_1 and S_2 states in solvent switch and match the order in the gas phase, hence all further calculations are performed exclusively in the gas phase.

Supplementary Table 1: Isoquinoline excitation energies in the gas-phase at the SCS-ADC(2)/aug-cc-pVQZ level of theory from the ground-state equilibrium geometry optimized at the SCS-MP2/cc-pVDZ level.

State	Transition	Energy / eV	f_{osc}	λ / nm
S_1	ππ*	4.26	0.0264	291
S_2	nπ*	4.81	0.0022	258
S_3	ππ*	4.90	0.0615	253

Supplementary Table 2: Isoquinoline excitation energies computed using COSMO to describe hexane solvation at the SCS-ADC(2)/aug-cc-pVQZ level of theory from the ground-state equilibrium geometry optimized at the SCS-MP2/cc-pVDZ level.

State	Transition	Energy / eV	f_{osc}	λ / nm
S_1	ππ*	4.23	0.0384	293
S_2	nπ*	4.82	0.0026	257
S_3	ππ*	4.86	0.0759	255

Supplementary Table 3: Quinoline excitation energies in the gas-phase at the SCS-ADC(2)/aug-cc-pVQZ level of theory from the ground-state equilibrium geometry optimized at the SCS-MP2/cc-pVDZ level.

State	Transition	Energy / eV	f_{osc}	λ / nm
S_1	ππ*	4.29	0.0261	289
S_2	nπ*	4.75	0.0024	261
S_3	ππ*	4.81	0.0584	258

Supplementary Table 4: Quinoline excitation energies computed using COSMO to describe hexane solvation at the SCS-ADC(2)/aug-cc-pVQZ level of theory from the ground-state equilibrium geometry optimized at the SCS-MP2/cc-pVDZ level.

State	Transition	Energy / eV	f_{osc}	λ / nm
S_1	ππ*	4.28	0.0368	290
S_2	nπ*	4.74	0.0029	262
S_3	ππ*	4.76	0.0792	260

Supplementary Table 5: Quinazoline excitation energies in the gas-phase at the SCS-ADC(2)/aug-cc-pVQZ level of theory from the ground-state equilibrium geometry optimized at the SCS-MP2/cc-pVDZ level.

State	Transition	Energy / eV	f_{osc}	λ / nm
S_1	nπ*	4.21	0.0034	294
S_2	ππ*	4.33	0.0268	286
S_3	ππ*	4.91	0.0436	252

Supplementary Table 6: Quinazoline excitation energies computed using COSMO to describe hexane solvation at the SCS-ADC(2)/aug-cc-pVQZ level of theory from the ground-state equilibrium geometry optimized at the SCS-MP2/cc-pVDZ level.

State	Transition	Energy / eV	f_{osc}	λ / nm
S_1	nπ*	4.24	0.0040	292
S_2	ππ*	4.30	0.0368	288
S_3	ππ*	4.83	0.0567	257

Supplementary Table 7: Quinoxaline excitation energies in the gas-phase at the SCS-ADC(2)/aug-cc-pVQZ level of theory from the ground-state equilibrium geometry optimized at the SCS-MP2/cc-pVDZ level.

State	Transition	Energy / eV	f_{osc}	λ / nm
S_1	nπ*	4.04	0.0040	307
S_2	ππ*	4.19	0.0913	295
S_3	ππ*	4.58	0.0344	271

Supplementary Table 8: Quinoxaline excitation energies computed using COSMO to describe hexane solvation at the SCS-ADC(2)/aug-cc-pVQZ level of theory from the ground-state equilibrium geometry optimized at the SCS-MP2/cc-pVDZ level.

State	Transition	Energy / eV	f_{osc}	λ / nm
S_1	nπ*	4.06	0.0046	305
S_2	ππ*	4.17	0.1254	297
S_3	ππ*	4.46	0.0448	278

Supplementary Table 9: 1,6-Naphthyridine excitation energies in the gas-phase at the SCS-ADC(2)/aug-cc-pVQZ level of theory from the ground-state equilibrium geometry optimized at the SCS-MP2/cc-pVDZ level.

State	Transition	Energy / eV	f_{osc}	λ / nm
S_1	ππ*	4.32	0.0387	287
S_2	nπ*	4.59	0.0002	270
S_3	nπ*	4.80	0.0040	258
S_4	ππ*	4.94	0.0420	251

Supplementary Table 10: 1,6-Naphthyridine excitation energies computed using COSMO to describe hexane solvation at the SCS-ADC(2)/aug-cc-pVQZ level of theory from the ground-state equilibrium geometry optimized at the SCS-MP2/cc-pVDZ level.

State	Transition	Energy / eV	f_{osc}	λ / nm
S_1	ππ*	4.29	0.0541	289
S_2	nπ*	4.62	0.0004	268
S_3	nπ*	4.83	0.0047	257
S_4	ππ*	4.92	0.0517	252

Supplementary Table 11: 1,8-Naphthyridine excitation energies in the gas-phase at the SCS-ADC(2)/aug-cc-pVQZ level of theory from the ground-state equilibrium geometry optimized at the SCS-MP2/cc-pVDZ level.

State	Transition	Energy / eV	f_{osc}	λ / nm
S_1	nπ*	4.31	0.0000	288
S_2	ππ*	4.33	0.0914	286
S_3	nπ*	4.82	0.0051	257
S_4	ππ*	5.10	0.0530	243

Supplementary Table 12: 1,8-Naphthyridine excitation energies computed using COSMO to describe hexane solvation at the SCS-ADC(2)/aug-cc-pVQZ level of theory from the ground-state equilibrium geometry optimized at the SCS-MP2/cc-pVDZ level.

State	Transition	Energy / eV	f_{osc}	λ / nm
S_1	ππ*	4.31	0.1244	288
S_2	nπ*	4.37	0.0000	284
S_3	nπ*	4.88	0.0062	254
S_4	ππ*	5.05	0.0743	246

2.2 Potential energy profiles to the S_1 state

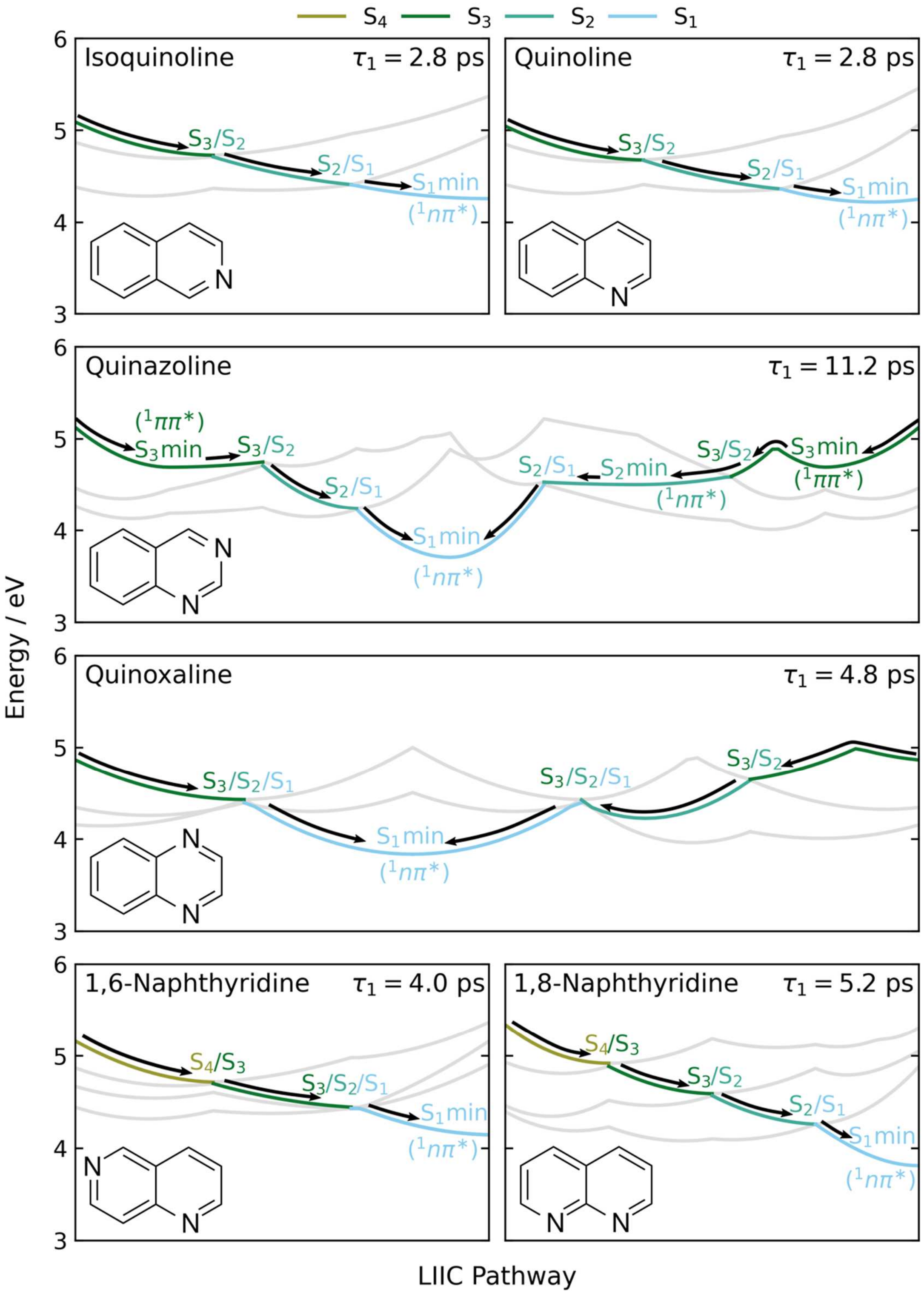

Supplementary Figure 5: Potential energy profiles of the photorelaxation pathway from S_3 (S_4 for 1,6- and 1,8-naphthyrdine) onto the S_1 surface, shown in Fig. 6 of the man text, with all excited states now shown. We show the non-active states along the pathways in light grey.

2.3 The S_2 surface in quinazoline

In addition to the pathway from the S_2 minimum to the S_1 minimum, there is another pathway present, which accesses the S_2/S_1 MECI on the left-hand side of Fig. 6 in the main text *via* TS12. Both these pathways from the S_2 minimum to the S_1 minimum are shown in Supplementary Fig. 6.

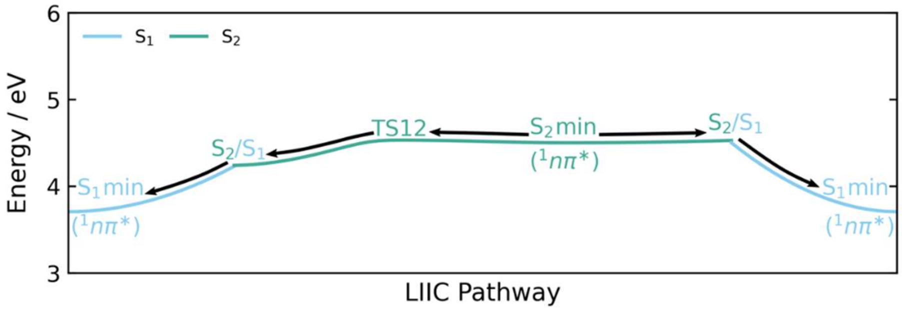

Supplementary Figure 6: Potential energy (PE) profile of the S_2 surface, in quinazoline, which leads to the S_1 ($^1n\pi^*$) minimum, from the S_2 ($^1n\pi^*$) minimum calculated SCS-ADC(2)/cc-pVDZ. The PE profiles have been interpolated between minima, the TS, and MECIs.

2.4 Triplet photorelaxation pathways

In Supplementary Fig. 7, we show the photorelaxation pathways from the S_1/T_2 MECPs to the T_1 minima in all six molecules. As mentioned in the main text, only isoquinoline, quinoline and quinoxaline have a T_2 minimum, however, in quinoxaline, the molecule will pass through the T_2/T_1 MECI before reaching this minimum, and hence, has been excluded from the plot in Supplementary Fig. 7.

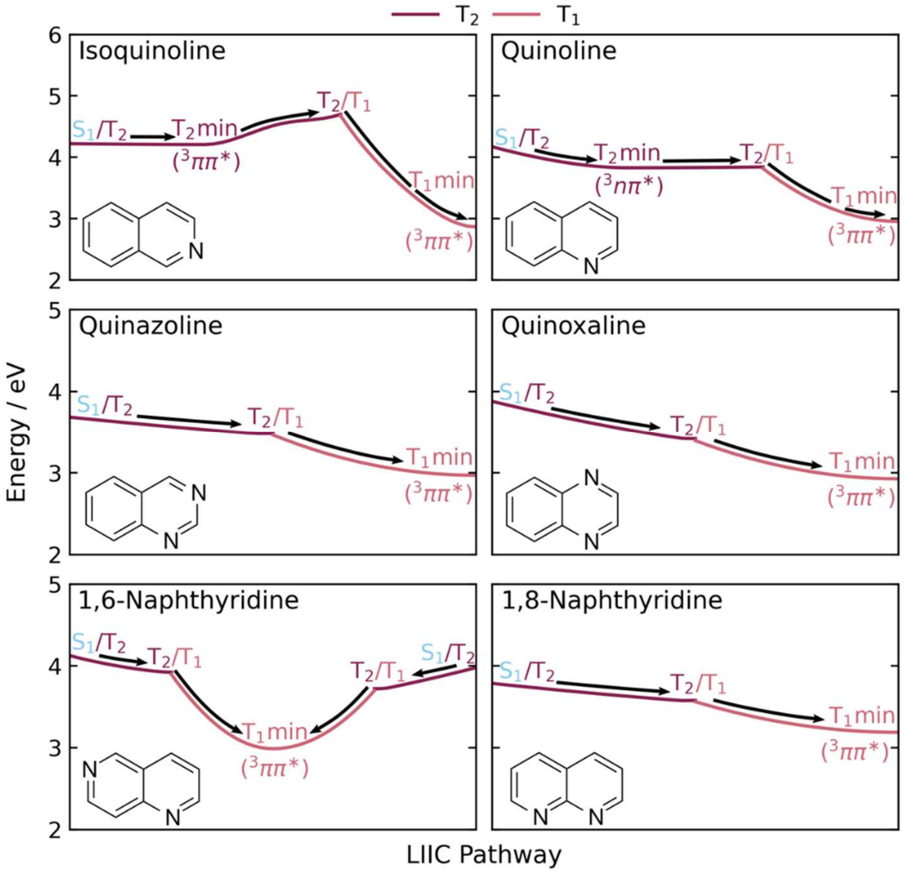

Supplementary Figure 7: Potential energy profiles of the photorelaxation pathways from the S_1/T_2 MECPs to the T_1 minimum, for all six molecules, computed using SCS-ADC(2)/cc-pVDZ. The profiles have been linearly interpolated between the MECPs, MECIs and excited-state minima. For clarity, only the active state involved along the pathway to the T_1 minimum is shown. The dominant electronic character of each excited state minimum is denoted in brackets.

Once on the T_1 surface, the six molecules are expected to stay around the T_1 minima as the T_1/S_0 MECPs are much higher than the minima, with values ranging from 0.48-0.83 eV dependent on the molecule. The T_1/S_0 MECPs all resemble the same geometries as the S_1/S_0 MECIs, where motion out of the plane occurs. The pathways from the T_1 minima to the T_1/S_0 MECPs, are shown in Supplementary Fig. 8. Although the spin-orbit coupling between the T_1 state and the ground-state cannot be calculated at the SCS-ADC(2)/SCS-MP2 level, the lifetime in the triplet manifold is proposed to be much

longer than the singlet manifold, due to the fact that the T_1/S_0 MECPs are less accessible compared to the S_1/S_0 MECIs, which rationalizes the longer than 50 ns lifetime.

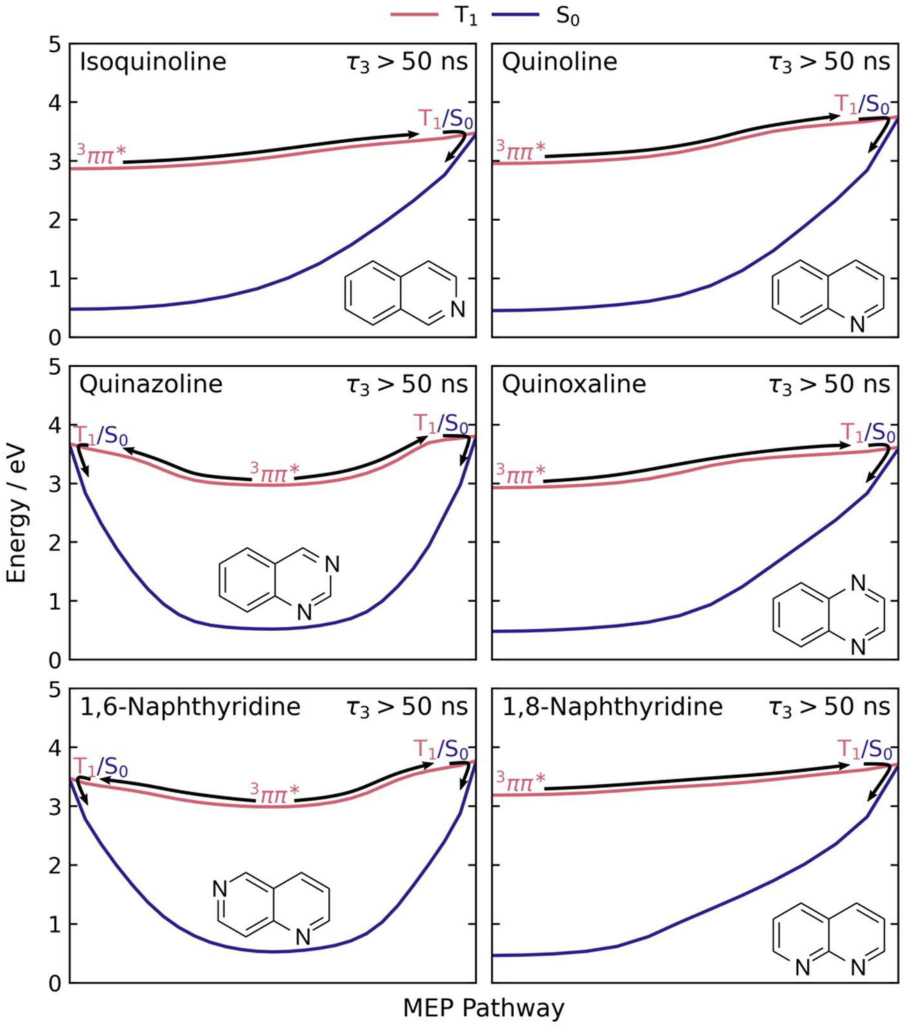

Supplementary Figure 8: Minimum energy pathways of the photorelaxation pathways from the T_1 ($^3\pi\pi^*$) minima to the T_1/S_0 MECPs, in all six molecules, calculated at the SCS-ADC(2)/cc-pVDZ level of theory.
