# Supplementary material for: Excited state dynamics of azanaphthalenes reveal opportunities for the rational design of photoactive molecules
Source: Commun Chem. 2025 Jan 9;8:7. doi: 10.1038/s42004-024-01403-z (PMC11717923; doi:10.1038/s42004-024-01403-z)
Supplement: Supplementary file 2 — Description of Additional Supplementary Files [file 42004_2024_1403_MOESM2_ESM.pdf]

Description of Additional Supplementary Files

File name- Supplementary Data 1

File description- Molecular geometries
